# Role of Kampo Medicine in Integrative Cancer Therapy

**DOI:** 10.1155/2013/570848

**Published:** 2013-08-22

**Authors:** Jun-ichi Yamakawa, Yoshiharu Motoo, Junji Moriya, Masao Ogawa, Hiroaki Uenishi, Sumiyo Akazawa, Toshiyuki Sasagawa, Matomo Nishio, Junji Kobayashi

**Affiliations:** ^1^Department of General Medicine, Kanazawa Medical University, 1-1 Daigaku Uchinada Kahoku District, Ishikawa 920-0293, Japan; ^2^Department of Medical Oncology, Kanazawa Medical University, Ishikawa 920-0293, Japan; ^3^Department of Anesthesia, Kanazawa Medical University, Ishikawa 920-0293, Japan; ^4^Department of Obstetrics and Gynecology, Kanazawa Medical University, Ishikawa 920-0293, Japan; ^5^Department of Pharmacology, Kanazawa Medical University, Ishikawa 920-0293, Japan

## Abstract

Clinical trials to date demonstrate that standard cancer treatments are currently the most efficient treatments for large numbers of cancer patients. Cancer treatments will increasingly require approaches that allow patients to live with cancer, by increasing their natural healing power and tumor immunity, as well as attenuating the progression of their cancers, instead of only attacking the cancer cells directly. Complementary and alternative medicine, including Kampo medicine, compensates for the drawbacks of western medicine by increasing patients' self-defense mechanisms. In Japan, clinicians who have studied both western medicine and Kampo treat cancer patients by fusing the two medical systems into a unitary one. The goal of the system is to assist the functional maintenance and recovery of the living body complex with the physical, mental, social, and spiritual balance, rather than addressing direct antitumor effects. In this review, we describe the usefulness of Kampo medicine, especially juzentaihoto, and outline the reports on evidence, in addition to the report on an attitudinal survey about the use of Kampo medicine in cancer treatment in Japan.

## 1. Limitations of Standard Cancer Treatment

Western medicine should be used in preference to Kampo medicine in the diagnosis and treatment of cancer. Medical diagnosis based on western medicine is essential to determine the exact degree of progression and malignancy of cancer [1–4]. Western medicine successfully treats many types of cancer, when the appropriate treatment is used. Clinical trials to date demonstrate that standard cancer treatments are currently the most efficient treatments for large numbers of cancer patients [5–8]. The current standard cancer treatments include surgery, chemotherapy, and radiation therapy. Reliable therapy has not been established yet for refractory cancer, including advanced or recurrent cancer. 

Advanced experimental therapies using special anticancer agents, radiation, immunotherapy, and gene therapy have been attempted. Radiation therapy, chemotherapy, and surgery are called “invasive treatments” [9–12]. The term “invasive” means “harmful to the body” as well as “attacking the cancer.” Aggressive treatment has the disadvantage of damaging normal tissue and reducing tumor immunity and physical strength. This is especially true in bone marrow cells or intestinal mucosal epithelia, in which the cell cycle is vigorous [13–18]. The tissues and organs of the whole body maintain order in their structure and function by incorporating nutrients and oxygen from the blood into cells, excreting waste, and repairing old and scar tissues. This is called “natural healing power” or “self-healing power.” Abnormal cells such as cancer cells are constantly produced in our body, but as long as the immune system is maintained properly, they are eliminated prior to growth or progression. This means that as long as the immune system is working properly, cancer is constantly being “cured,” without any treatment.

Cancer treatments will increasingly require approaches that allow patients to live with cancer, by increasing their natural healing power and tumor immunity, as well as attenuating the progression of their cancers, instead of attacking the cancer cells [19–21]. Complementary and alternative medicine, including Kampo medicine [22–25], compensates for the drawbacks of western medicine by increasing the body's healing power and resistance [26–29]. The term “multidisciplinary treatment of cancer” refers to treatment that appropriately utilizes a combination of treatments such as standard treatment, advanced experimental treatments, medical care to enhance the anticancer and healing power of the body, and palliative care, depending on the state of the cancer patient. The goal of holistic treatment, or integrated care, is not only to kill cancer cells but also to address the healing power and resistance of the body. Kampo treatment can provide very useful therapeutic options to achieve holistic therapy and multidisciplinary treatment for cancer [30–36].

## 2. Surveys of the Use of Kampo in Cancer Treatment in Japan

Three papers investigating the use of Kampo in cancer treatment in Japan were published in 2012. Takeda et al. [37] conducted a self-reported questionnaire on Kampo medicine involving a total of 476 patients with gynecologic cancers. Anxiety was assessed using the State-Trait Anxiety Inventory. It was confirmed that 22.9% of the women had used Kampo medicine. Kampo users were more likely to have had chemotherapy and were more likely to have experienced uncomfortable adverse effects of cancer treatment. Kampo users were more likely to believe that Kampo offers relief of symptoms with less adverse effects and that Kampo is more effective than western medicine. Kampo users expressed stronger attitudes in regard to taking Kampo medicine. Multiple regression analysis revealed that chemotherapy (RR, 1.82; 95% CI, 1.14–2.91), lower state anxiety (RR, 0.76; 95% CI, 0.58–1.00), and higher trait anxiety (RR, 1.46; 95% CI, 1.11–1.92) were independently associated with Kampo use. The study showed that approximately one-fourth of Japanese gynecologic cancer patients take Kampo medicine. Kampo users made more favorable comments on Kampo medicine than nonusers. The authors concluded that the psychological characteristics of individual patients are one of the factors that can influence the usage of Kampo.

Iwase et al. [38] conducted a cross-sectional self-administered anonymous questionnaire among 549 physicians working in palliative care teams at 388 core cancer treatment hospitals and 161 certified medical institutions that have palliative care units (PCUs). Valid responses were obtained from 311 physicians (56.7%) who were evenly distributed throughout the country without significant geographical biases. Kampo medicines were prescribed for controlling cancer-related symptoms by 64.3% of the physicians. The symptoms treated with Kampo medicines were numbness/hypoesthesia (*n* = 99, 49.5%), constipation (*n* = 76, 38.0%), anorexia/weight loss (*n* = 72, 36%), muscle cramps (*n* = 71, 35.5%), and languor/fatigue (*n* = 64, 32.0%). Regarding open issues about prescription, 60.7% (*n* = 173) of the physicians raised the issue that the dosage forms need to be better devised. The authors concluded that more evidence from clinical studies is needed to increase the clinical use of Kampo medicines and that the action mechanisms of Kampo should be clarified through laboratory research. 

Ito et al. [39] conducted a nationwide survey to investigate the use of Kampo medicine by Japanese physicians in the core cancer treatment hospitals designated by the national Ministry of Health, Labour and Welfare. Among the 900 physicians surveyed, 92.4% reported having prescribed Kampo medications, and 73.5% of those physicians reported having prescribed Kampo medications for cancer patients. Despite this high percentage of usage and the finding that 9.7% of the physicians considered Kampo medications to be harmful, only 23.1% of the physicians expressed high expectations of the efficacy of Kampo medicine in tumor suppression and exertion of an immunostimulatory action. In contrast, many of cancer patients expressed the belief that Kampo can suppress tumor growth. The authors concluded that further research on the efficacy and safety of Kampo medicine in cancer treatment is warranted to resolve this discrepancy between patients' and physicians' expectations.

## 3. The Role of Kampo Medicine in Multidisciplinary Treatment of Cancer

The Kampo medicine approach is to prevent cancer by emphasizing the body's defense mechanism and natural healing power [22–25]. The existence of even early stage cancer indicates that the healing system is already in functional decline. The fundamental approach of Kampo treatment is to remove obstacles to healing, compensate for deficiencies, and consider the required combination of crude drugs. In western medicine, ideas aimed at activation and nourishing of tissue function are scant, whereas Kampo medicine considers these ideas as the most important treatment strategy. Kampo medicine recognizes that diseases are processes of struggling between individual-specific resistance power and external force of robing those power (e.g., virus). And the former directly determines the occurrence, progression, and outcome of disease. Sanity is a Kampo concept including all of the antidisease substances in the living body, which in modern medicine equates to natural healing power, comprising the mechanisms of self-defense, homeostasis, immune surveillance, and tissue repair. The immune system, antioxidant actions to prevent harm by active oxygen, and the tissue repair system also basically correspond to the concept of sanity in Kampo medicine. In Kampo cancer treatment, we try to treat dysfunction of the body that leads to cancer progression and also try to enhance body function to regain sanity. This is a core characteristic of Kampo treatment of cancer.

The present position of Kampo medicine in the medical treatment of cancer in Japan allows patients to access western and Kampo medical treatments simultaneously. Kampo medicine [22–25] is a unique medical system that originated from ancient China, was gradually imported to Japan, and has been improved and refined by many excellent physicians since the Edo period. Most Kampo preparations (Japanese traditional herbal medicines) are available as extract formulations, which are greatly different from the herbal medicine practiced in China, Taiwan, and Korea. Four ethical Kampo extract formulations were approved in 1967 in Japan. Since then, the number of ethical Kampo extract formulations covered by health insurance has grown to 148. Japan's universal health insurance system [40, 41] does allow for simultaneous access to traditional Kampo preparations and western medicines. However, physicians in Japan cannot be licensed without passing a board examination on western medicine, which means that patients in this country receive health care with a high degree of safety. This is another factor that distinguishes the health care system in Japan from other countries. In Japan, physicians who have studied western medicine and Kampo medicine practice these approaches in their medical treatment of cancer with the aim of fusing eastern and western medicine into a unitary medical system (unlike the dual medical systems in China or Republic of Korea).

The goal of the system is to assist the functional maintenance and recovery of the living body complex, a host incorporating a nutrient state, mental balance, and so forth, rather than addressing direct anticancer efficacy. The present condition and stance of Kampo medicine in the medical treatment of cancer in Japan clearly diverges from development of an anticancer herbal medicine and formulation of an antitumor herbal tablet.

## 4. Juzentaihoto: A Typical Kampo Formula for Cancer Treatment

**Figure 1:**
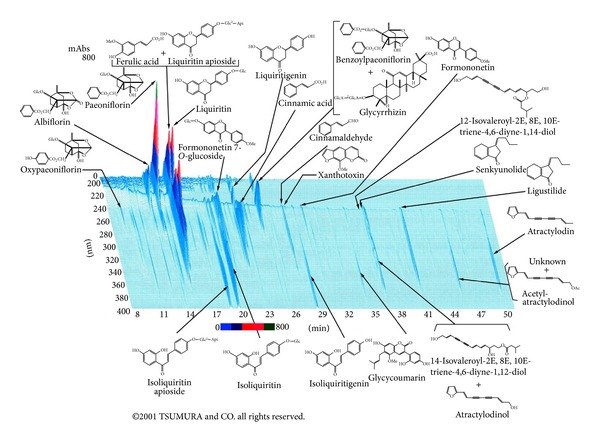
3D-HPLC pattern of TJ-48 juzentaihoto.

Juzentaihoto is an effective Kampo medicine for promoting restoration of physical strength after surgery and alleviating adverse effects of anticancer drugs or radiation therapy. While there are many other useful Kampo medicines, this discussion focuses on juzentaihoto.  Table 1 and Figure 1 show juzentaihoto's composition and constituent analysis. Juzentaihoto is indicated for the relief of declined constitution after recovery from disease, fatigue and malaise, anorexia, perspiration during sleep, cold limbs, and anemia. Important precautions, (1) when this product is used, patient's “*sho*” (constitution and symptoms) should be taken into account. Patient's progress should be carefully monitored, and if no improvement in symptoms or findings is observed, continuous treatment should be avoided. (2) Since this product contains Glycyrrhiza, careful attention should be paid to the serum potassium level, blood pressure, and so forth, and if any abnormality is observed, administration should be discontinued. (3) When this product is coadministered with other Kampo preparations (Japanese traditional herbal medicines), attention should be paid to the duplication of the contained crude drugs.

Juzentaihoto itself has also been reported to prevent cancer occurrence and recurrence, as well as to suppress metastasis. A major report found that juzentaihoto stimulated activation of pluripotent hematopoietic stem cells in irradiated mice [42]. It has also been reported to have an antiangiogenic action in malignant glioma. There have been studies of its actions in suppressing carcinogenesis and metastasis [43–48]. 

Juzentaihoto's biological activity has also been widely reported. Among these reports are studies that found that it activates macrophages, enhances antibody production, induces cytokine production, and has other immunoenhancement actions, in addition to protection against disturbance of myelopoiesis and against immune suppression in anticancer drug and radiation therapy [49].

Muraishi et al. [50] examined the effect of juzentaihoto on immunological functions and antitumor activity in old mice. Juzentaihoto increased the number of T cells remarkably and NK cells slightly in the aged mice, while a significant increase was not observed in young mice.

Treatment with juzentaihoto increased NK activity in both young and old mice. Therefore, the combination of IFNs with juzentaihoto may provide a means to increase the therapeutic potential of IFNs and to decrease their toxicity for the treatment of metastatic renal cell carcinoma. Juzentaihoto increased regulatory activities in T cells by decreasing Foxp3 (+) Treg populations in advanced pancreatic cancer patients [51]. This effect can lead to immunoaugmentation for various combination therapies. Genetic analysis using microarrays has recently been used to indicate its effects in germ-free mice [52]. Ogawa et al. also report that myelosuppression due to TS-1 in mice may be improved with coadministration of juzentaihoto [53]. It is often used for adjunct therapy to cancer therapy as well as the main clinical goals of improving patients' quality of life and alleviating the side effects of anticancer agents and radiation therapy, anorexia, fatigue, and malaise and reduced physical strength following illness. Tokushima University Hospital introduced Kampo medicine therapy to improve general malaise and the various side effects caused by chemotherapy and radiation therapy, which is used at the hospital for planocellular carcinoma of the uterine cervix [54]. The hospital reported that Kampo medicines are allowed for prolongation of the lives of cervical cancer patients. Satou and Arakawa investigated whether Kampo therapy based on traditional approaches is useful or not for inhibition of hepatic cell carcinogenesis (HCC) and reported that therapies based on traditional approaches are useful for HCC in chronic type C liver disease [55].

## 5. Summary

Several aspects of Kampo treatment in Japan have been introduced here with respect to holistic care, integrative therapy, and multidisciplinary treatment of cancer patients. The Kampo medicine approach is to control cancer by bringing out the natural healing power inherent to living bodies and emphasizing body's defense mechanism. In western medicine, ideas aimed at activation of organ functions and nourishment are scant, whereas Kampo medicine regards enhancement of self-defense mechanisms as the most important strategy. Integrative cancer therapy using Kampo medicine is expected to develop further in Japan.

## Figures and Tables

**Table 1:** Composition of juzentaihoto.

Description	Juzentaihoto extract granules for ethical use	
Composition	7.5 g of juzentaihoto extract granules contains 5.0 g of a dried extract of the following mixed crude drugs	
	JP Astragalus Root	3.0 g
	JP Cinnamon Bark	3.0 g
	JP Rehmannia Root	3.0 g
	JP Peony Root	3.0 g
	JP Cnidium Rhizome	3.0 g
	JP Atractylodes Lancea Rhizome	3.0 g
	JP Japanese Angelica Root	3.0 g
	JP Ginseng	3.0 g
	JP Poria Sclerotium	3.0 g
	JP Glycyrrhiza	1.5 g
	Inactive ingredients	
	JP Magnesium Stearate	
	JP Lactose Hydrate	
